# Innovation Glass-Ceramic Spray Deposition Technology Improving the Adhesive Performance for Zirconium-Based Dental Restorations

**DOI:** 10.3390/ijms232112783

**Published:** 2022-10-24

**Authors:** Chien-Ming Kang, Dan-Jae Lin, Sheng-Wei Feng, Cheng-Yuan Hung, Shogo Iwaguro, Tzu-Yu Peng

**Affiliations:** 1 Huayi Dental Laboratory, Taipei 10491, Taiwan; 2 Department of Biomedical Engineering, College of Biomedical Engineering, China Medical University, Taichung 40402, Taiwan; 3 School of Dentistry, College of Dentistry, China Medical University, Taichung 40402, Taiwan; 4 Research Center of Digital Oral Science and Technology, College of Oral Medicine, Taipei Medical University, Taipei 11031, Taiwan; 5 School of Dentistry, College of Oral Medicine, Taipei Medical University, Taipei 11031, Taiwan; 6 Department of Dentistry, National Yang Ming Chiao Tung University, Taipei 11221, Taiwan; 7 Division of Dental Technician, Department of Clinical Practice and Support, Hiroshima University Hospital, Hiroshima 734-8551, Japan

**Keywords:** zirconia, lithium disilicate glass-ceramic, spray deposition, thermocycling, shear bond strength, dental restorations

## Abstract

Glass-ceramic spray deposition (GCSD) is a novel technique for coating lithium disilicate (LD) glass-ceramics onto zirconia through simple tempering steps. GCSD has been proven to improve the bonding of zirconia to resin cement, but the effect of etching time on GCSD and the long-term durability of the bond achieved remain unknown. The effects of air abrasion with aluminum particles (ABB) and air abrasion (GAB) or etching with 5.0% hydrogen fluoride (HF) for 20, 60, 90, and 120 s (G20, G60, G90, and G120) on the resin cement–zirconia bond were studied. LD was included as a control (LDG). The microstructure, sub-micron roughness, wettability, and phase changes of samples were analyzed. After resin cement was bonded to zirconia, half of the samples were subjected to thermocycling (5000 cycles at 5–55 °C). The bond strengths of the samples were determined in shear bond strength (SBS) tests (n = 10 per group). An LD structure can be formed on zirconia after GCSD and proper etching processes, which result in high roughness and a hydrophilic nature. GCSD and HF etching significantly improved SBS, with G90 and G120 samples with pre- or post-thermocycling exhibiting SBS values comparable to those of LDG (*p* > 0.760). The surface characteristics of the LD layer are influenced by the etching time and affect the SBS of the bond of zirconia to resin cement. HF etching for 90–120 s after GCSD results in zirconia with SBS and bond durability comparable to LD.

## 1. Introduction

Zirconia restorations have attracted the attention of clinical dentistry because of their excellent mechanical properties, high transparency, and natural appearance [1,2,3]. Traditional esthetic dental restoration involves fusing porcelain with metal, but in the last decade, porcelain fused with zirconia and full zirconia restorations have increasingly been used [4,5,6]. Computer-aided design/manufacturing (CAD/CAM) is widely accepted by dentists and dental technicians because of its precision and efficiency [7,8]. As the requirements of the fully digital workflow, accompanied by oral scanning, have increased, the demand for multilayer transparency and gradient-color “monolithic zirconia restorations” that do not require veneering has increased [8,9,10]. The main weaknesses of zirconia are its unsatisfactory clinical bonding performance and evidence from long-term follow-up studies showing that the bond between zirconia and dental cement is poorer than that of lithium disilicate (LD) glass-ceramics [11,12]. The main reason for this is that zirconia only has a crystal phase, not a glassy one. The crystal phase structure gives zirconia good mechanical properties, but the surface is challenging to roughen and therefore is not prone to adhere other materials. Compared with LD, which contains a glassy phase, the surface of zirconia is not easy to roughen or etch with acid to create micromechanical interlocking effects. Additionally, a ceramic primer cannot be used to form a covalent bond with the zirconia crystalline structure. Thus, the primary goal of continuous improvement in the clinical practice of prosthodontics and operative dentistry is determining how to treat the zirconia surface to modify its physical or chemical properties to improve the bond between zirconia and dental cement [13,14,15,16,17].

Several methods are commonly used to improve the bonding of zirconia in clinical dentistry. Air abrasion using alumina oxide particles improves micromechanical interlocking at the zirconia surface, which can improve bonding [14,16,18]. Shimoe et al. [18] evaluated various air-abrasion conditions and reported that the best results are achieved with 50-µm alumina particles and a jet pressure of 2–3 bars. However, the results reported for air abrasion have been mixed, and air abrasion may also have side effects and pose risks of physical damage to zirconia [19,20]. Immersion of zirconia in a high concentration of a strong acid, such as sulfuric acid, has been attempted to achieve the effect of overall surface roughening; however, this method is hazardous in clinical dental operations, and no apparent improvement of the bond strength has yet been reported in the literature [13,14,21]. A primer containing 10-methacryloxydecyl dihydrogen phosphate (MDP) has been used to smear the zirconia surface, creating a P-O-Zr ionic bond that promotes bonding of zirconia to resin [22,23]. This method is convenient and cost-effective and can effectively increase the wettability and roughness of the zirconia [24,25], thereby improving the bonding durability of zirconia restorations [22,23,26]. Some scientists have recently attempted using plasma [2,27,28], laser [3,15,17], and fusion sputtering [29,30] for surface treatment. These techniques improve the zirconia–resin bond strength, but require unique or expensive equipment, involve complicated operation processes, and are difficult to standardize and impractical for dental practices.

The gold standard for surface treatment of zirconia is to combine air abrasion and an MDP-based primer to increase the micromechanical interlocking and chemical bonding of zirconia to dental resin. However, how to achieve bond strength and durability that are comparable to those achievable with traditional dental ceramics remains unclear. Glass-ceramic spray deposition (GCSD) is a surface treatment technique that can improve the bond effectiveness of zirconia restorations [20]. GCSD involves spraying glass-ceramic powders on zirconia and then sintering at a proper temperature. A thin dense LD layer can then be formed. This layer will mechanically bond to the zirconia surface and increase the bonding of zirconia to dental cement. GCSD is applied to strengthened zirconia, not in the green stage. Thus, it does not affect the physical properties of zirconia during the high-temperature crystallization–strengthening–shrinkage process, nor does it affect the final strength and material properties after sintering. After GCSD treatment, a glass-ceramic layer is coated on the zirconia surface. The only follow-up clinical operations required are etching, priming, and cementation. The high-density and uneven molecular distribution characteristics of GCSD make it possible for zirconia to achieve a better bond strength comparable to traditional glass-ceramics.

The GCSD technique has been proven to improve the bond strength between zirconia and resin cement, but the effects of etching time on the bond strength and durability after GCSD treatment are not yet fully understood [20]. One objective of this research was to evaluate the effects of different hydrogen fluoride (HF) acid etching times on the zirconia–resin bond strength. A second objective was to evaluate whether the GCSD technique can increase the long-term performance of the zirconia–resin bond. The achievement of these two objectives is expected to contribute to the ultimate goal of establishing clinical guidelines for GCSD. The two null hypotheses considered in this study were that (1) the etching time after GCSD does not affect zirconia–resin bond performance and that (2) the GCSD does not improve the durability of the zirconia–resin bond.

## 2. Results

### 2.1. Surface Microstructure and Roughness

**Figure 1:**
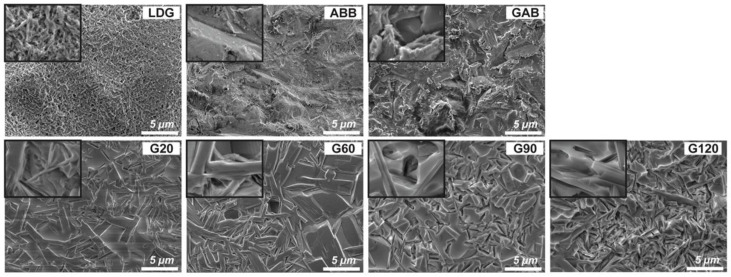
Images of microstructure of sample surface observed via FE-SEM (Scale bar = 5 μm. The width of the small enlargement in each image is 1.25 μm).

**Figure 2:**
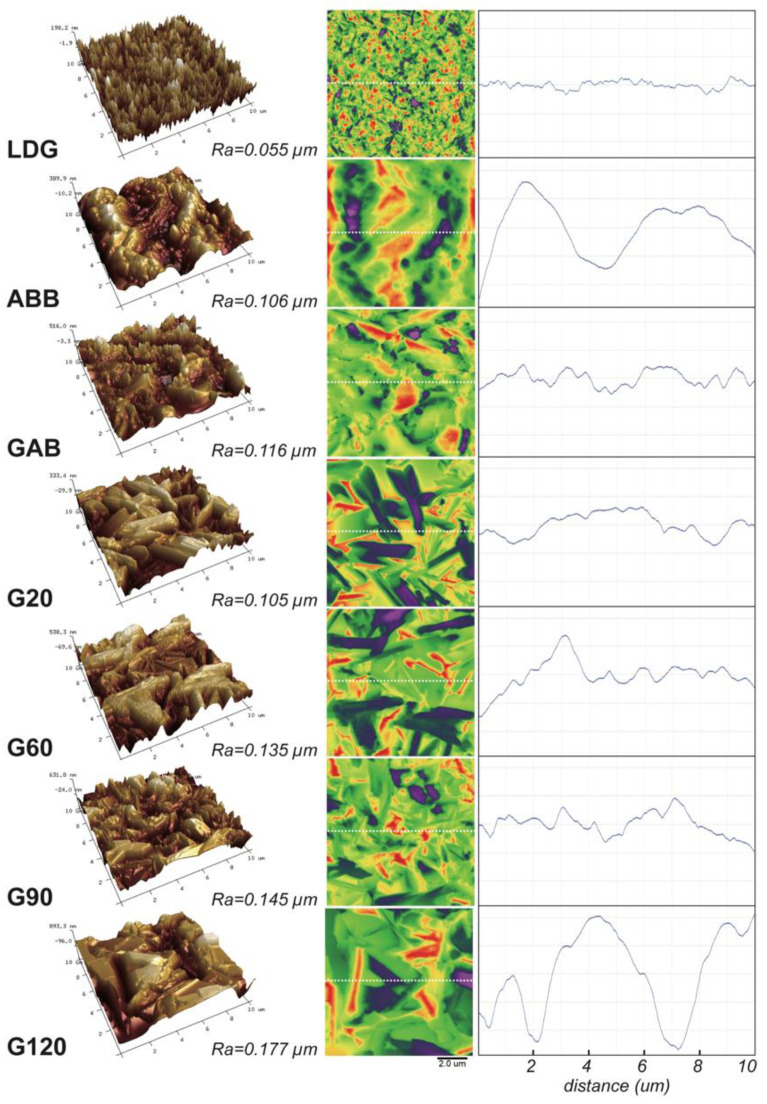
10 × 10 μm^2^ atomic force microscopy (AFM) images of all samples. The sub-micron roughness (Ra) of the surfaces obtained from the flattened surfaces are also shown. The linear profile is perpendicular to the peak between two valleys selected along the white dotted line in each corresponding two-dimensional map.

The microstructure of the testing samples is presented in Figure 1. LGD showed removal of the glassy phase and exposure of the lithium disilicate crystals. ABB exhibited irregular, uneven, and rough characteristics. The surface of GAB combined with LD crystal and the disordered structure caused by air abrasion. The other GCSD groups (G20, G60, G90, and G120) exhibited a dense crystal structure of rod-like lithium metasilicate and interlocking needle-shaped LD crystal. Notably, the acid etching time affected the amount of the glassy phase, and a lower amount of the glassy phase was associated with more LD crystal exposure. The Ra results are illustrated in Figure 2. The highest Ra was 0.18 µm for G120, and the lowest was 0.06 µm for LDG. The Ra was found to increase with increasing acid etching time. The combined treatment of GCSD and air abrasion (GAB) resulted in high Ra values. Linear profiles (Figure 2) revealed that the surface of LDG was relatively flat, whereas the surfaces of the five GCSD groups were relatively rough and with an evident heave.

### 2.2. Surface Hydrophobicity and Surface Free Energy

**Figure 3:**
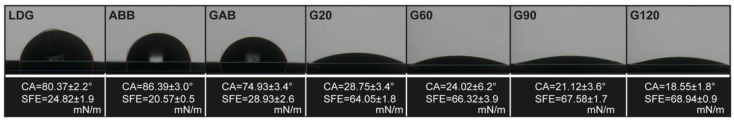
Contact angles (CA) and surface free energy (SFE) were measured on sample surfaces after different pretreatment methods were applied.

Figure 3 illustrates the CA and SFE results for the samples. The LDG and ABB groups were hydrophobic, with a CA larger than 80°. Among the GCSD groups, the GAB had a slightly smaller CA of 75°, and after etching, all of the samples exhibited hydrophilic surfaces (CA < 30°). The group treated with GCSD accompanied by HF etching exhibited a significantly higher SFE than the LDG, ABB, and GAB groups (*p* < 0.001). No statistically significant differences were detected between groups with different etching times (*p* > 0.363), between LDG and ABB (*p* = 0.301), or between LDG and GAB (*p* = 0.516).

### 2.3. Surface Phase Structure

**Figure 4:**
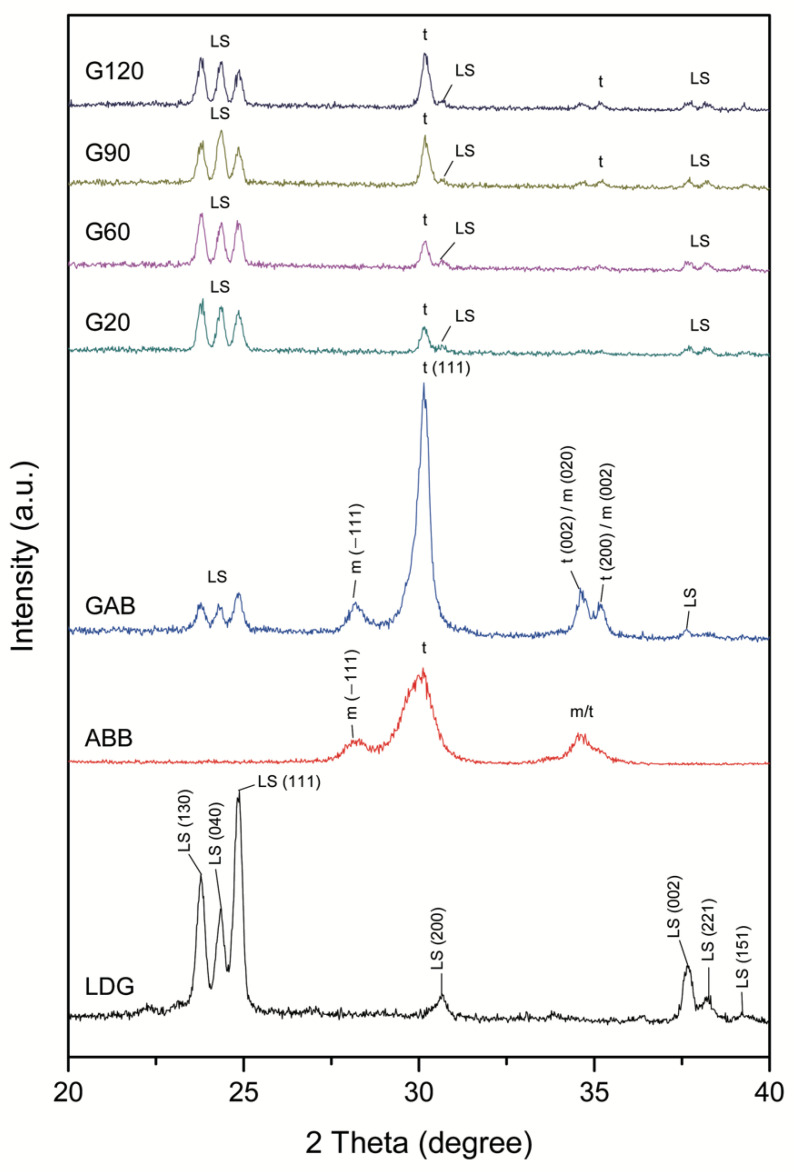
Grazing-incident X-ray diffraction (XRD) patterns show the surface phase structures of glass spray coating-modified Y-TZP (GAB, G20, G60, G90, and G120) compared to air-abrasion (ABB) and the LD glass-ceramic control group (LDG). Here, *m* indicates the XRD pattern of monoclinic zirconia (*m*-ZrO_2_), *t* indicates tetragonal zirconia (*t*-ZrO_2_), and LS indicates LD (Li_2_Si_2_O_5_).

Figure 4 shows the grazing-incidence XRD patterns of seven different surfaces. The XRD diffraction peaks of LDG at 23.78°, 24.34°, 24.88°, 30.72°, 37.68°, 38.28°, and 39.28° were assigned to corresponding crystal plane of 1 3 0, 0 4 0, 1 1 1, 2 0 0, 0 0 2, 2 2 1, and 1 5 1, respectively, for the LD (Li_2_Si_2_O_5_) crystallization (JCPD #14-0322). For ABB, broadened and shifted peaks of tetragonal zirconia (*t*-ZrO_2_, 1 1 1), monoclinic zirconia (*m*-ZrO_2_, −1 1 1), and *m/t*-ZrO_2_ were observed (0 0 2/0 2 0 and 2 0 0/0 0 2; *t*-ZrO_2_: JCPD #17-0923 and *m*-ZrO_2_ JCPD #37-1484). For GAB, the main reflecting peak was at 31.5° of *t*-ZrO_2_, and diffraction peaks of *m*-ZrO_2_ were also present, with diffraction peaks at 28.2°, 34.3°, and 35.3° corresponding to −1 1 1, 0 0 2/0 2 0, and 2 0 0/0 0 2, respectively. Notably, the glass-ceramic coating on the Y-TZP surface transformed from an amorphous phase to Li_2_Si_2_O_5_. Hence, a reflecting peak for Li_2_Si_2_O_5_ (LS) was also detected. For the G20, G60, G90, and G120 groups, the reflection peaks of LS become more significant with increasing etching time, and the reflecting peak of *t*-ZrO_2_ also became more distinct.

### 2.4. Adhesive Properties

**Figure 5:**
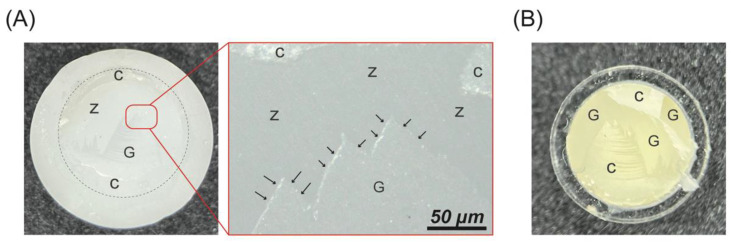
Representative debonded fracture surface of mixture failure on the sample side (**A**) and resin-cement side (**B**). Z: zirconia, G: glass-ceramic coatings, C: resin cement. Arrows point to interface debonding.

Table 1 presents the SBS and observed failure modes of the test groups. Before thermocycling, the highest SBS was 20.5 MPa for G120, while the lowest was 8.6 MPa for GAB. However, the LDG (*p* > 0.999) and G90 (*p* = 0.269) groups exhibited no statistically significant differences from the G120 group. After 5000 thermocycles, the LDG and G120 groups exhibited significantly higher SBS than the AAB, GAB, G20, and G60 groups (*p* ≤ 0.001). The LDG, G90, and G120 groups were comparable in terms of SBS (*p* > 0.760). All groups exhibited significant SBS reduction after thermocycling (*p* < 0.001). The LDG, AAB, and G120 groups exhibited better bonding durability. All groups that did not undergo thermocycling exhibited mixture failure predominantly, except AAB, for which 50% of the failures were classified as adhesive failures. The G90 and G120 groups exhibited 100% mixture failures. After 5000 thermocycles, the failure mode was predominantly mixture failure, except for the AAB and G20 groups, for which it was predominantly adhesive failure. Note that the GAB and G120 groups exhibited 100% mixture failures. Figure 5 presents the representative debonded fracture surface of mixture failure.

## 3. Discussion

The GCSD technique was developed for creating thin and dense LD glass-ceramic coatings on zirconia surfaces to overcome the inadequacy of the bonding performance of zirconia without changing the phase structures or physicochemical properties of zirconia. Sundfeld et al. [31] explained that the HF etching mechanism on LD glass-ceramic consists of removing the glassy matrix, which enables the HF to dissolve the Si-O bonds in the glass-ceramic. The exposed LD crystals become sites for micromechanical interlocking with resin cement. Peng et al. [20] confirmed that GCSD accompanied by proper HF etching can significantly improve the strength of the zirconia–resin cement bond. Sundfeld et al. [32] reported that a higher concentration of HF increased the removal of the glassy matrix, leading to a higher depth of dissolution of the glassy matrix and thus an enhanced bond strength. However, higher concentrations of HF pose a hazard. The optimal HF etching concentration for GCSD was determined to be 5% [20]. Veríssimo et al. [33] recommended etching for 20 to 60 s for LD glass-ceramics, and Peng et al. [20] suggested that 100 s is optimal for GCSD.

One aim of this research was to assess the effect of the etching time (20, 60, 90, or 120 s) on SBS. Results indicate that the longer the etching agent stays on the zirconia surface, the more deeply the glassy phase is eroded and the more obviously the surface topography changes. From the change in the surface phase structure (Figure 4), the characteristic peak of zirconia *t*-ZrO_2_ (1 1 1, 0 0 2, and 2 0 0) becomes clear because of the level of etching. The microstructure of the four GCSD groups (G20, G60, G90, and G120) comprises LD crystals exhibiting a long-needle shape embedded in a glassy matrix, and the degree of crystallinity increases with increasing etching time (Figure 1). These results are consistent with those of several previous studies of the crystal morphology of LD glass-ceramics [34,35]. The AFM images (Figure 2) indicate that as the acid etching time increases, the profile shape becomes more stereoscopic, and the waviness, asperity, and finish of the surface become more obvious. Surface topography changes also affected the sub-micron roughness and the lubrication regime of zirconia. The surface analysis results show that as the acid etching time increases: (1) Ra increases (Figure 2), making the contact area between resin cement and zirconia larger [16]; (2) the SFE increases (Figure 3), with the creation of functional groups and reactive sites and improvement of the adhesiveness of zirconia [36]; and (3) the wettability increases (Figure 3), facilitating spreading of the resin cement across the entire surface and achievement of a good bond [37]. These changes in surface characteristics increase the SBS of the zirconia–resin cement bond with increasing etching time (Table 1). Thus, the first null hypothesis—that the etching time after GCSD does not affect the bonding between zirconia and resin—is rejected.

Air abrasion makes the zirconia surface disordered and irregular, which facilitates interlocking between the resin cement and zirconia and increases the SBS. Skienhe et al. [38] suggested that air abrasion could lead to zirconia experiencing a tetragonal-to-monoclinic (*t-m*) phase transformation, which would decrease the fracture strength, surface damage, and microcrack formation. The current study examined air abrasion for one group (AAB), and the experimental results were consistent with those reported in the literature (Figure 4). A broadened and shifted peak of *t*-ZrO_2_ (1 1 1) was observed after air abrasion. Air abrasion after GCSD treatment (GAB) results in the appearance of multi-characteristic peaks such as *m*-ZrO_2_, *t*-ZrO_2_, and LS. However, the prominent peak was zirconia because of the LD glass-ceramic coating being destroyed by Al_2_O_3_ particle abrasion and then debonding, so only a tiny amount of LD glass-ceramic remained on the surface (Figure 2). The LD glass-ceramic coating produced by the GCSD mainly contained SiO_2_, which can form Si-O bonds and enhance the degree of bonding through a reaction between the Si-OH groups on the zirconia surface and those in the resin cement [39]. However, after the LD glass-ceramic coating was destroyed by air abrasion, the loss of these chemical bonds resulted in a significantly lower SBS (8.6 MPa) than for the other experimental groups (*p* < 0.05). Thus, air abrasion after GCSD treatment is not recommended.

The LDG samples contained LD glass-ceramic block (Figure 4), the structure of which consists of randomly oriented small and interlocking needle-shaped crystals (Figure 1) [34,35]. The AFM image (Figure 2) of the surface of the LDG group shows that its topography was relatively smooth, but effective chemical bonding was achieved after priming; thus, a desirable SBS of 18.6 MPa was obtained (Table 1). The gold standard of surface treatments, represented by the ABB group, had a significantly lower SBS (12.5 MPa) than the LDG group (*p* < 0.01) (Table 1). These results are consistent with reports in the literature that the bonding performance of zirconia is inferior to that of LD [11,12]. Compared with that of the ABB group, the SBS was significantly improved after GCSD treatment, especially for the groups treated with HF acid etching for 90 s (19.1 MPa) or 120 s (20.5 MPa), whose SBS values were comparable to those of the LDG group (Table 1). This can also be explained in terms of the failure mode. At either 0 or 5000 thermocycles, the failures in the G90 and G120 groups were predominantly mixed failures (Figure 5), indicating that the resin cement and LD glass-ceramic coatings achieved good interlocking, significantly improving SBS.

Thermal cycling was used as an artificial aging method to evaluate bond durability in the current research. All groups exhibited significant SBS reductions (Table 1) after artificial aging (*p* < 0.01), consistent with previous studies [15,23,28]. Thermal cycling contributes to debonding of the resin cement from the surface of the zirconia because of their different thermal expansion coefficients and the hygroscopic natures of resin cement and zirconia [18]. Compared with the ABB group (59.5%), GCSD followed by more than 20 s of HF etching improved bond durability. The etching time was negatively correlated to SBS reduction. When the etching time increased to 120 s (G120), the reduction decreased to 51%. GAB exhibited the lowest percentage reduction in SBS (50.6%) among all GCSD groups because of its originally lower SBS at 0 thermocycles. Thus, the second null hypothesis that GCSD treatment does not improve the durability of the zirconia–resin bond is rejected.

Note that the LDG group exhibited the lowest reduction (43.9%), which might be because of its higher SFE and lower Ra, making it difficult for water molecules to enter the interface of the material. However, the precise reasons still need to be examined in the future. Based on the results of this study, 5% HF etching for 90 to 120 s after GCSD treatment is recommended to achieve the strongest and most durable bond between resin cement and zirconia.

## 4. Materials and Methods

Table 2 presents the study design and trial steps. Disk-shaped samples 10 mm in diameter and 2.5 mm thick were prepared using a dental milling machine (Cameo 250i, Aidite Technology Co., Ltd., Qinhuangdao, China). The samples were distributed into seven groups: one group of LD glass-ceramic samples (denoted LDG) and six groups are zirconia samples. The LDG group was the control group. The LDG samples were etched with 5.0% HF (IPS Ceramic Etching Gel, Ivoclar Vivadent AG, Schaan, Liechtenstein) for 20 s. In the blasting group (denoted by AAB), the samples were air-blasted with 50-μm aluminum particles (Cobra, Renfert GmbH, Hilzingen, Germany) under a pressure of 3 bars using a dental blaster machine (Basic Eco, Renfert GmbH, Hilzingen, Germany). The other five groups were subjected to GCSD (Biomic LiSi connector, Aidite Technology Co., Ltd., Qinhuangdao, China) using the method described by Peng et al. [20]. After application of the GCSD treatment, the GAB group was subjected to air abrasion under the same conditions as described above, and the other four groups were etched with 5.0% HF for 20, 60, 90, or 120 s (denoted by G20, G60, G90, and G120, respectively).

The surface morphologies of the samples were assessed using a field emission scanning electron microscope (FE-SEM; JEOL JSM-7800F Prime, JEOL Ltd., Tokyo, Japan) in secondary electron mode. Each sample’s sub-micron roughness (Ra) was analyzed via atomic force microscopy (AFM; Dimension Icon VT-1000, Bruker Taiwan Co., Ltd., Hsinchu, Taiwan) over a 10 × 10 μm^2^ area. Contact angles (CAs) were measured to investigate the wettability of the samples using a CA analyzer (Phoenix Mini, Surface Electro Optics Co., Ltd., Gyeonggi-do, Korea). Each reported CA value was calculated based on ten independent attempts. The surface free energy (SFE) was calculated by the Girifalco–Good–Fowkes–Young model [40], based on the CA data obtained as described above. The phase changes of the samples were studied using a high-resolution X-ray diffractometer (XRD; D8 SSS, Bruker Taiwan Co., Ltd., Hsinchu, Taiwan) under grazing-incident diffraction conditions.

Z-Prime Plus (Bisco, Inc., Schaumburg, IL, USA) was used to prime the AAB group, and Monobond N (Ivoclar Vivadent AG, Schaan, Liechtenstein) was used to prime the other six groups. A plastic tube (6.0 mm in diameter and 2.0 mm in height) was then attached to each sample with a piece of double-sided polyethylene adhesive tape to define the bonding area. Resin cement (Variolink N, Ivoclar Vivadent AG, Schaan, Liechtenstein) was flowed into the plastic tube and light-cured for 10 s (Bluephase Style Curing Light, Ivoclar Vivadent AG, Schaan, Liechtenstein), and any residual cement was removed. One hour was allowed to elapse to ensure that the cementation was complete. One-half of the samples were immersed in distilled water at 37 °C for 24 h. The remaining samples were placed in a self-assembling thermocycling apparatus and cycled between 5 and 55 °C in distilled water for 5000 cycles with a dwell time of 30 s and a transfer time of 15 s, in accordance with the ISO 10477 standard [41]. A universal testing machine (JSV-H1000, Japan Instrumentation System Co., Ltd., Nara, Japan) was used to perform SBS testing on specimens that did and did not undergo thermocycling (n = 10). The SBS testing was conducted at a constant crosshead speed of 1 mm/min, and the shear force was applied to the interface of the sample (ISO 10477). The load at which debonding occurred was recorded. After the SBS testing, the debonded surface was observed with a dental microscope (10×). Failures were classified as adhesive failure if the bond failed at the glass-ceramic or zirconia surface, cohesive failure if the failure occurred in the resin cement, and mixture failure if both adhesive and cohesive failure occurred. Representative structures of the residual surface adhesive were examined using an optical microscope (Motic BA210; Motic Medical Diagnostic Systems, Co., Ltd., Kowloon, Hong Kong).

The sample size was calculated based on a power analysis with a power of 1.0 and an α error of 0.05, which enabled clinically justified recommendations. Normality was assessed primarily by the Shapiro–Wilk test. Statistical comparisons of the test results for the various groups were performed using a one-way analysis of variance (ANOVA) with Tukey’s honestly significant difference (HSD) tests for multiple comparisons. The statistical analyses were performed using SPSS 19 (IBM, New York, NY, USA) and Prism 9.0 (GraphPad Software, San Diego, CA, USA). The threshold for significant differences was set to *p* < 0.05 unless otherwise specified.

## 5. Conclusions

This study aimed at investigating the effect of etching on GCSD treatment effectiveness and at evaluating whether zirconia can achieve a sufficiently durable bond with resin cement after GCSD treatment. The acid etching time significantly affects the microstructure, sub-micron roughness, and wettability of the LD layer, which improves the SBS. Particularly for the G90 and G120 groups, SBS values comparable to those for LD were achieved even after artificial aging by thermal cycling. GCSD accompanied by 5% hydrogen fluoride acid etching for 90–120 s and appropriate chemical priming is recommended to produce the best surface physicochemical structure to enhance the bonding performance between zirconia and resin cement, expanding the usable range and prolonging the life of zirconium-based dental restorations.

## Figures and Tables

**Table 1:** Mean values of SBS (MPa) and failure modes for each group.

Groups	0 Thermocycles				5000 Thermocycles				S	Reduction
	Mean ± SD	A	M	C	Mean ± SD	A	M	C		
LDG	18.6 ± 2.7 ^a^	3	7	0	10.4 ± 1.5 ^A^	4	6	0	S	43.9%
AAB	12.5 ± 2.1 ^b^	5	5	0	5.1 ± 1.1 ^B^	7	3	0	S	59.5%
GAB	8.6 ± 1.7 ^c^	2	8	0	4.3 ± 1.3 ^B^	2	8	0	S	50.6%
G20	11.7 ± 1.9 ^b^	4	6	0	4.5 ± 1.4 ^B^	6	4	0	S	61.8%
G60	15.9 ± 2.5 ^d^	2	8	0	6.5 ± 1.8 ^B,C^	2	8	0	S	59.2%
G90	19.1 ± 3.0 ^a^	0	10	0	8.8 ± 1.5 ^A,C^	1	9	0	S	53.6%
G120	20.5 ± 2.1 ^a^	0	10	0	10.1 ± 1.2 ^A^	0	10	0	S	51.0%

SD: standard deviation. Within a column, different letters indicate statistically different groups (*p* < 0.05): A: adhesive failure, M: mixture of cohesive and adhesive failures, C: cohesive failure. S: significant difference between 0 and 5000 thermocycles (*p* < 0.05). Reduction: the bond strength reduction rate from 0 to 5000 thermocycles.

**Table 2:** Study design and trial steps.

Prepare 30 lithium disilicate (LD) glass-ceramic (Cameo Dental Glass Ceramics) and 180 zirconia (Superfect Zir) disk-shaped samples via dental CAD/CAM system (Cameo 250i).						
Grind the samples flat using 600-grit silicon carbide abrasive papers, clean them ultrasonically with deionized water, and air-dry them.						
Distribute the samples into seven groups (n = 30)						
LD Glass-Ceramic	Zirconia (Y-TZP)					
**LDG**	**AAB**	Glass-Ceramic Spray Deposition (GCSD) Treatment (Biomic LiSi Connector)				
		**GAB**	**G20**	**G60**	**G90**	**G120**
5% HF (IPS Ceramic Etching Gel) etching for 20 s	air abrasion with 50-μm aluminum particles under 3-bar pressure for 10 s		5% HF etching for 20 s	5% HF etching for 60 s	5% HF etching for 90 s	5% HF etching for 120 s
Surface characterization analysis (n = 10): FE-SEM, AFM, CA, SFE, XRD.						
Bonding according to the following procedures.						
Priming with Monobond N	Priming with Z-Prime (Bisco)	Priming with Monobond N (Ivoclar Vivadent)				
Attachment of a plastic tube to each sample with a piece of double-sided polyethylene adhesive tape.						
Flowing resin cement (Variolink N) into the plastic tube, light-curing, and removing residual cement.						
Division of each group (n = 20) as follows:						
Immersion of one-half of the samples (n = 10) in distilled water at 37 °C for 24 h without thermocycling.			Interrupted thermocycling of one-half of the samples (n = 10) between 5 and 55 °C for 5000 cycles with a dwell time of 30 s (ISO 10477).			
Shear bond strength (SBS) testing using a universal testing machine (JSV-H1000, Japan Instrumentation System) at a crosshead speed of 1 mm/min (ISO 10477).						
Observation of the failure mode (adhesive, cohesive, mixture failure) with a dental microscope and recording of representative structures via an optical microscope (BA210, Motic).						
Statistical analysis (IBM SPSS and Prism)						

## Data Availability

Not applicable.
